# Induced pluripotent stem cell-derived human macrophages as an infection model for *Leishmania donovani*

**DOI:** 10.1371/journal.pntd.0011559

**Published:** 2024-01-02

**Authors:** Lore Baert, Serena Rudy, Mélanie Pellisson, Thierry Doll, Romina Rocchetti, Marcel Kaiser, Pascal Mäser, Matthias Müller

**Affiliations:** 1 Swiss Tropical and Public Health Institute (SwissTPH), Basel, Switzerland; 2 University of Basel, Basel, Switzerland; 3 Novartis Institutes for Biomedical Research, Novartis Pharma AG, Basel, Switzerland; Bernhard Nocht Institute for Tropical Medicine, Hamburg, Germany, GERMANY

## Abstract

The parasite *Leishmania donovani* is one of the species causing visceral leishmaniasis in humans, a deadly infection claiming up to 40,000 lives each year. The current drugs for leishmaniasis treatment have severe drawbacks and there is an urgent need to find new anti-leishmanial compounds. However, the search for drug candidates is complicated by the intracellular lifestyle of *Leishmania*. Here, we investigate the use of human induced pluripotent stem cell (iPS)-derived macrophages (iMACs) as host cells for *L*. *donovani*. iMACs obtained through embryoid body differentiation were infected with *L*. *donovani* promastigotes, and high-content imaging techniques were used to optimize the iMACs seeding density and multiplicity of infection, allowing us to reach infection rates up to 70% five days after infection. IC_50_ values obtained for miltefosine and amphotericin B using the infected iMACs or mouse peritoneal macrophages as host cells were comparable and in agreement with the literature, showing the potential of iMACs as an infection model for drug screening.

## Author summary

Yearly, up two million people in poverty-stricken areas contract leishmaniasis, a disease caused by parasites of the genus *Leishmania*. When an infected sandfly takes a blood meal, *Leishmania* parasites enter the host where they are taken up by macrophages. Inside the macrophage, *Leishmania* parasites establish a niche where they can proliferate. The infection often leads to disability and can result in death. Moreover, the drugs currently available are unsatisfactory due to toxic side effects, high expenses or difficulties in usage. Drug screening assays that are currently used for compound screening often rely on mouse peritoneal macrophages. We have generated human induced pluripotent stem cell derived macrophages and used these as new host cells for *Leishmania donovani* in the testing of anti-leishmanial compounds. This model has many advantages. For one, it allows us to work with human cells, mimicking the natural infection more closely than murine cells. Secondly, it allows us to obtain bigger batches of uniform cells for screening campaigns. Finally, this approach aligns with the principles of the 3Rs, replacing the use of animals for cultivation of *Leishmania* and drug screening purposes.

## Introduction

Up to one million people in nearly 100 endemic countries contract leishmaniasis every year, rendering the infection one of the most important neglected tropical diseases [1]. Leishmaniasis is caused by protozoan parasites of the genus *Leishmania*, which are transmitted to a mammalian host when an infected phlebotomine sandfly takes a blood meal [2]. Upon entry into the host, the parasites are phagocytized, mostly by macrophages. Strikingly, *Leishmania* parasites not only survive but even thrive in the hostile environment of the macrophages, where they establish a niche in the parasitophorous vacuole [3,4]. *Leishmania* is characterized by two distinct life cycle stages. Inside the sandfly, *Leishmania* exists as its promastigote form, which is recognizable by a single, long flagellum protruding from the anterior end of an elongated cell body. Once inside the macrophages, the acidic environment and higher temperature trigger the differentiation of the promastigotes to amastigotes, which are smaller and only have a rudimental flagellum [4,5].

More than 20 known *Leishmania* species have the ability to infect humans, causing different forms of leishmaniasis [6]. The most severe form is visceral leishmaniasis (VL), an infection of the internal organs, in particular the liver and the spleen. VL is caused mainly by *L*. *donovani* and *L*. *infantum* and is almost always fatal if left untreated [7]. While there are effective drugs on the market, they all have severe drawbacks such as a high cost, impracticability of use, adverse side effects or rising drug resistance [8]. Thus, there is an urgent need for new and better treatments options.

The intracellular localization of *Leishmania* complicates the search for new active molecules. Compounds are most relevantly tested against the intracellular amastigotes [9]. Macrophages obtained from various sources have been used to study host-parasite interaction and anti-leishmanial drug efficacy. These include bone marrow-derived murine macrophages [10], peritoneal murine macrophages (PMMs) [11,12], human peripheral blood monocyte-derived macrophages (hMDMs) [13], and immortalized monocyte-like cell lines such as THP-1 cells [13,14]. Each of these has some disadvantages. The primary macrophages are incompatible with high-throughput screening as their isolation is time-consuming, the yields are low, and there is a large variation between batches [15]. On the other hand, the malignant origin of the monocyte-like cell lines such as THP-1, makes them a less physiologically accurate model for investigating host-parasite interaction [16]. A promising alternative to these macrophage populations are iPS-derived macrophages [15,17–19].

In 2012, Yamanaka discovered that somatic cells can be dedifferentiated into a pluripotent state by overexpression of the transcription factors Oct4, Sox2, Klf4 and c-Myc [20]. Since then, induced pluripotent stem cells (iPSCs) have been differentiated into dozens of different cell types, including hepatocytes [21,22], neurons [23,24], endothelial cells [25] and macrophages [18,19,26]. iPS-derived macrophages (iMACs) highly resemble their counterparts isolated from human donors, making them a favoured system compared to transformed cell lines like THP1, which often harbour multiple uncharacterized genetic mutations [27]. Working with human iMACs versus primary murine cells is not only more practical but also more ethical as it reduces the use of laboratory animals. In addition, the use of human iMACs is more relevant since mice have a different immune profile than humans. For example, even in susceptible strains such as the BALB/c mice, visceral leishmaniasis is not fatal, which is in sharp contrast to infection in humans [28,29]. Finally, it is relatively easy and quick to generate large quantities of genetically identical iMACs. All these advantages make iMACs an attractive model for drug screening.

In this study, we made use of human iMACs to establish a new infection model for *L*. *donovani*. After optimizing the culture condition for infectibility with late-stage promastigotes the two reference drugs, miltefosine and amphotericin B, were tested for their anti-leishmanial activity in iMACs and compared to the standard model using PMMs.

## Materials & Methods

### Ethics statement

Isolation of peritoneal murine macrophages from mice were conducted in accordance with the strict guidelines set out by the Swiss Federal Veterinary Office, under the ethical approval of license number #BL/524 (Animal Protection Authority, Canton Baselland).

### Media

*Leishmania culture medium*: Equal parts SM and SDM-79 medium [30,31] (pH 7.4) supplemented with 10% heat-inactivated FCS and 2 μg/mL hemin. *mTeSR Plus medium*: mTeSR Plus Basal Medium (pH 7.4) (STEMCELL Technologies) supplemented with mTeSR Plus 5x Supplement and 1% penicillin/streptomycin (Gibco). *Complete RPMI medium*: RPMI 1640 Glutamax (pH 7.4) (Gibco) supplemented with 10% heat-inactivated FCS (Gibco), 1% sodium pyruvate (Gibco), 1% penicillin/streptomycin, 25 mM HEPES (Gibco), 0.055 mM β-mercaptoethanol (Gibco). *MDM medium*: DMEM (pH 7.4) (Gibco) supplemented with 10% FBS, 1% Glutamax (Gibco), 0.055 mM β-mercaptoethanol, 1x MEM Non-Essential Amino Acids (Gibco) and 1% penicillin/streptomycin. *X-Vivo 15 medium*: X-Vivo 15 (pH 7.4) (Lonza) supplemented with 1% Glutamax, 0.055 mM β-mercaptoethanol and 1% penicillin/streptomycin.

### *L*. *donovani* culturing

*Leishmania donovani* (MHOM/ET/67/HU3, ATCC-50127) promastigotes were maintained at 27°C in *Leishmania* culture medium. Cultures were passaged three times a week (1,100), maintaining a concentration in the order of 10^5^ parasites/ mL. To obtain metacyclic promastigotes, the cultures were incubated for five days until reaching stationary growth phase with a concentration of ca. 5 x 10^7^ parasites/ mL.

### iPS cell culturing

Female human WT29 iPS cells were derived from cell line AG092429 obtained from the NIA Aging Cell Repository at the Coriell Institute for Medical Research. The male iPS line WT94 was generated from GM23394 also obtained from Coriell. Stem cells were plated on a LN511-coated surface (BioLamina) and amplified in mTeSR Plus medium. During the first 24 h after seeding, the medium was supplemented with 10 μM ROCK Inhibitor (Y-27632). The medium was changed every 24h and the cells were passaged every three days (1/30). For passaging, the cells were washed with PBS and detached by incubation with TrypLE (Gibco) for 5 min at 37°C.

### iMAC differentiation

On day one, iPS cells were resuspended in mTeSR Plus medium supplemented with 10 μM ROCK Inhibitor at a concentration of 60,000 cells/ml. The iPS cells were distributed to BIOFLOAT 96 well plates (FaCellitate) at 100 μL/well. On day two, 100 μL mTeSR Plus medium supplemented with 100 ng/mL BMP4, 40 ng/mL hSCF and 100 ng/mL VEGF was added to each well. On day three, 100 μL of medium was replaced with fresh mTeSR Plus medium supplemented with 50 ng/mL BMP4, 20 ng/mL hSCF and 50 ng/mL VEGF. On day five, embryoid bodies (EBs) measuring 400 to 700 nm in diameter were apparent. These were resuspended in MDM medium supplemented with 100 ng/mL hm-CSF (human macrophage colony stimulating factor) and 25 ng/mL IL3. EBs of two 96 well plates were then plated in a T75 flask coated with 0.1% gelatine in PBS. On day 8, the medium was replaced with X-Vivo 15 medium supplemented with 100 ng/mL hm-CSF and 25 ng/mL IL3. Every three to four days, two-thirds of the medium were replaced with fresh medium. After three weeks, EBs start to secrete iMACs of the unpolarized M0-state which were harvested from the supernatant. At the peak of iMAC production, op to 10 million iMACs can be isolated each week. After ca. three months, production of iMACs started to slow down. Incubation of the cells was done at 37°C, 5% CO_2_.

### iMAC polarization

After harvesting, iMACs were centrifuged for 5 min at 200g and resuspended in Complete RPMI medium supplemented with 40 ng/mL hm-CSF and seeded in 96 well plates. For polarization to M1 or M2a, 50 ng/mL IFNγ or 50 ng/mL IL-4 was added to the medium, respectively. The polarization was done for 48h or five days. Growth factors were maintained in the medium during the entire assay.

### iMAC characterisation using flow cytometry

iMACs were either directly harvested from the EB culture or detached by incubation with TrypLE for 5 min at 37°C following polarization. iMACs were then washed with PBS plus 2% FBS and incubated for 15 min at RT with 50 ul/mL Human TruStain FcX Blocking solution (BioLegend) in PBS + 2% FBS. Next, iMACs were stained for 1h on ice with mouse anti-human antibodies CD14-PE/ AF488, CD16-FITC, CD45-PE/ FITC, CD68-FITC, CD80-PE/ FITC, CD86-AF488, CD163-FITC, CD206-AF488 and the corresponding IgG1κ or IgG2b isotype controls (Invitrogen). Staining was followed by two washing steps in PBS + 2% FBS. When the samples were measured immediately, they were resuspended in 400 μL PBS + 2% FBS before adding 10 μL propidium iodide (PI) (10 mg/ mL, Eurogentec) for 2 min. Otherwise, iMACs were fixated for 15 min at RT in BD Cytofix buffer (BD Biosciences) and washed twice before being resuspended in 400 μL PBS + 2% FBS. The Attune NxT Acoustic Focusing Cytometer was used for measuring the samples (2.0 x 10^4^ events/sample). The data were analysed using FlowJo software.

### Murine cell culturing and seeding

Two days prior to harvesting of PMMs, CD1 mice (Charles River Germany) were inoculated with 0.5 mL starch solution (Fluka/ Sigma 85645). To harvest the cells, mice were euthanized and 10 mL of RPMI medium was injected into the peritoneal cavity using a 25G needle. Next, PMMs were extracted from the peritoneal cavity using a 22G needle. The PMMs were centrifuged 10 min at 200g and resuspended in RPMI medium containing 1% Mäsermix [32] complemented with 15% RPMI medium containing growth factors obtained through seven days of cultivating the LadMac cell line. Subsequently the PMMs were seeded in T75 flasks and incubated 37°C, 5% CO2. After three days, the PMMs were washed with EBSS and trypsinized for 20s using 0.05% trypsin. Using a cell scraper, the PMMs were fully detached and collected in complete RPMI medium before seeding in flat bottom cell culture microplates (uClear, Greiner Bio-One, Cell Star) for infection.

### hMDM culturing and seeding

Buffy coats were obtained from Bern Blood Centre SRK. Peripheral blood mononuclear cells were gained through Ficcoll gradient centrifugation. Monocytes were subsequently isolated using the EasyStep Human monocytes isolation kit (#19359, StemCell technologies). Purified monocytes were then resuspended in complete RPMI medium plus 40 ng/mL hm-CSF at a concentration of ca. 1 x 10^6^ cells/ mL. Two mL of the suspension was seeded in each well of a 6-well cell culture plate. Plates were incubated at 37°C and 5% CO_2_ for five days to allow the differentiation of monocytes to macrophages. Medium was changed every other day. After five days, cells were detached by incubation with 10 mM EDTA in PBS for 15 min at RT and collected in complete RPMI medium before seeding in flat bottom cell culture microplates (uClear, Greiner Bio-One, Cell Star) for infection.

### Infection with *L*. *donovani* and drug treatment

On day one, iMACs, hMDMs or PMMs were seeded at various densities in flat bottom cell culture microplates (uClear, Greiner Bio-One, Cell Star) in 100 μL Complete RPMI medium plus 40 ng/mL hm-CSF per well. The outer wells of the plates were not used and filled with PBS. On day three, the medium was aspirated. Late-stage promastigotes were centrifuged (15 min at 800 g), resuspended in 100 μL fresh Complete RPMI medium plus 40 ng/mL hm-CSF per well, and added to the macrophages at different MOIs. On day four, extracellular parasites were washed away twice with medium before 100 μL fresh Complete RPMI medium + 40 ng/mL hm-CSF per well was added to the macrophages. When drugs were used during the assay, they were added at this point. Either miltefosine (M5571, Sigma-Aldrich) resuspended in H_2_O or amphotericin B (A2411, Sigma-Aldrich) resuspended in DMSO were added to the medium at various concentrations (final concentration of DMSO <0.05%). On day eight, the macrophages were fixed and stained. Macrophages were always incubated at 37°C, 5% CO_2_.

### Fixation and staining

Macrophages were fixed with 4% formaldehyde for 20 min at RT in the dark (Sigma). After fixation, the cells were washed three times with PBS.

### DAPI and AF488-phalloidin stain

Macrophages were permeabilized with 0.1% Triton X-100 diluted in PBS for 90s. Afterwards, the cells were washed once with PBS. Next, the macrophages were stained with 100 μL of 5 μg/mL DAPI in PBS for 10 min at RT in the dark. Another washing step was performed with PBS before staining with 0.033 μM AF488 phalloidin (BioStatus) for 30 min at RT in the dark. After staining, the macrophages were washed three times with PBS. The wells were filled up with PBS and the plates were stored at 4°C.

### DRAQ5 stain

Macrophages were stained with 50 μL of 5.0 μM DRAQ5 (BioStatus) in PBS for 20 min at RT in the dark. After staining, the macrophages were washed three times with PBS. The wells were filled up with PBS and the plates were stored at 4°C.

### LAMP-1 stain

iMACs were permeabilized with methanol for 20 min at -20°C. Afterwards, cells were incubated for 1h in 0.1% Triton X100 and 2% BSA (Sigma Aldrich) diluted in PBS. This was followed by overnight incubation at 4°C with 1:200 anti- LAMP-1 (D401sm #15665, Life Technologies). The next day, the iMACs were washed 3 x 5 min with PBS and incubated for 1h with 1:1000 goat anti-mouse IgG-AF488 (A11001, Invitrogen). Afterwards the cells were washed 3 x 5 min with PBS again. The wells were filled up with PBS and the plates were stored at 4°C.

### Imaging and analysis

Infection assays were either imaged using the ImageXpress Micro XLS High Content Imager (Molecular Devices) or the CV7000 High Content Imager (Yokogawa), depending on availability. Between 9 to 16 images per well were taken with a 20x objective lens, ensuring at least 13% surface coverage. MetaXpress (Molecular Devices) or CellPathfinder (Yokogawa) software was used for automatic analysis of the images. The nuclei, cell bodies and intracellular dots were identified with a traditional image analysis approach using object segmentation based on a fluorescence intensity threshold (S1 Fig). iMACs stained with LAMP-1 antibody were imaged using confocal microscopy. Images were taken with a 40x objective lens using the LSM900 Airyscan (ZEISS) and analysed using the ZEN 3.2 software (ZEISS). For the calculation of the percentage of infected macrophages and the number of intracellular parasites per infected macrophage, the counts were corrected by subtracting the false positive signal calculated from an uninfected negative control sample. When performing drug assays, an untreated positive control sample was included and the percentage of infected macrophages and number of intracellular parasites per macrophage were corrected with the negative control and normalized by dividing the values with the corrected positive control value and multiplying by 100:

$$Leishmania\;per\;iMAC\;(\%)=\frac{\#Dots\;per\;iMAC\;(infected;treatd)-\#Dots\;per\;iMAC\;(uninfected)}{\#Dots\;per\;iMAC\;(infected;\;untreated)-\#Dots\;per\;iMAC\;(uninfected)}*100$$

$$Infected\;iMACs\;(\%)\;=\;\frac{\%\;positive\;iMACs\;(infected;treated)-\%\;positive\;iMACs\;(uninfected)}{\%\;positive\;iMACs\;(infected;untreated)-\%\;positive\;iMACs\;(uninfected)}*100$$

### Statistics

Data analysis was done using GraphPad Prism 9.5.1 software. All data are shown as mean ± SD calculated from a minimum of three independent experiments. IC_50_ values of compounds were calculated using non-linear regression to a sigmoidal dose-response curve. Statistical analysis was done using ANOVA tests and t-tests. P-values < 0.05 were considered statistically significant.

## Results

### Generation of iPS-derived macrophages

**Fig 1:**
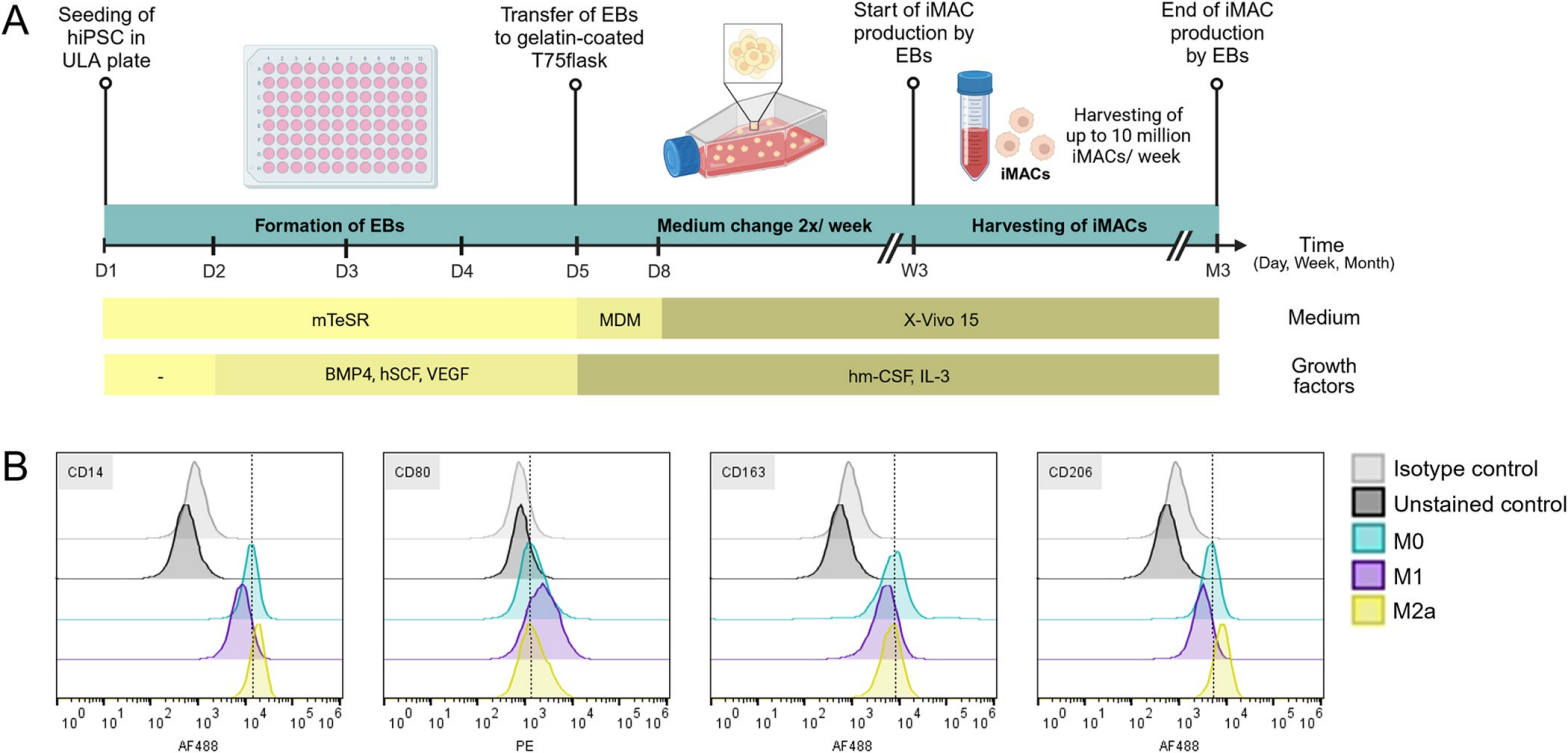
Differentiation of iPS cells to iMACs followed by 48h polarization. A) Schematic overview of the differentiation process of iPS cell to macrophage (iMAC) via the formation of embryoid bodies (EBs). Created with BioRender.com B) iMACs were polarised to the M0 (blue), M1 (IFNγ, purple) or M2a (IL-4, yellow) subtypes for 48h. Flow cytometry histograms show the expression of the cell surface receptors CD14, CD80, CD163 and CD206. Expression is compared to an unstained (black) and an isotype (grey) control. The dotted line marks the intensity peak of the M0 iMACs. The iMAC population was gated based on the FSC-A/ SSC-A plot, followed by gating of the single cells based on the FSC-A/ FSC-H plot and gating of live cells based on the BL2/ FSC-A plot.

iMACs were generated according to the published protocol of van Wilgenburg *et al* [18]. Two to three weeks after inducing the formation of embryoid bodies (EBs) from human iPS cells (line WT29), the EBs started to produce iMACs of the M0-like subtype in the supernatant expressing CD14, CD16, CD45, CD68, CD80, CD163 and CD206 (Figs 1A and S2). To test the plasticity of iMACs they were polarized with IFNγ or IL-4 into either the inflammatory M1 or anti-inflammatory M2a subtypes, respectively. A two vs. five-day treatment was tested to evaluate whether a shorter polarization time suffices. Polarization success was assessed by measuring the expression of the subtype-specific macrophage markers CD80 (M1), CD163 (M2) and CD206 (M2) (Figs 1B and S3). As expected, CD14, a marker for cells of the myeloid lineage, was highly expressed over all conditions. The expression level for CD80 showed was slightly elevated in M1 iMACs but was almost undetectable in other conditions. While CD163 was expressed in all subtypes, expression was lower in M1 iMACs compared to the M0 and M2a subtypes. Finally, M2a polarized iMACs show the highest expression of CD206. There was no striking difference in expression patterns for the M1 and M2 specific CD80, CD163 and CD206 markers between iMACs that had been polarized for two vs. five days suggesting a shorter polarization time suffices (Figs 1B and S3). This demonstrates that iMACs can response to inflammatory stimuli like primary macrophages [15].

### Determination of optimal experimental conditions for infection of iMACs with *L*. *donovani*

**Fig 2:**
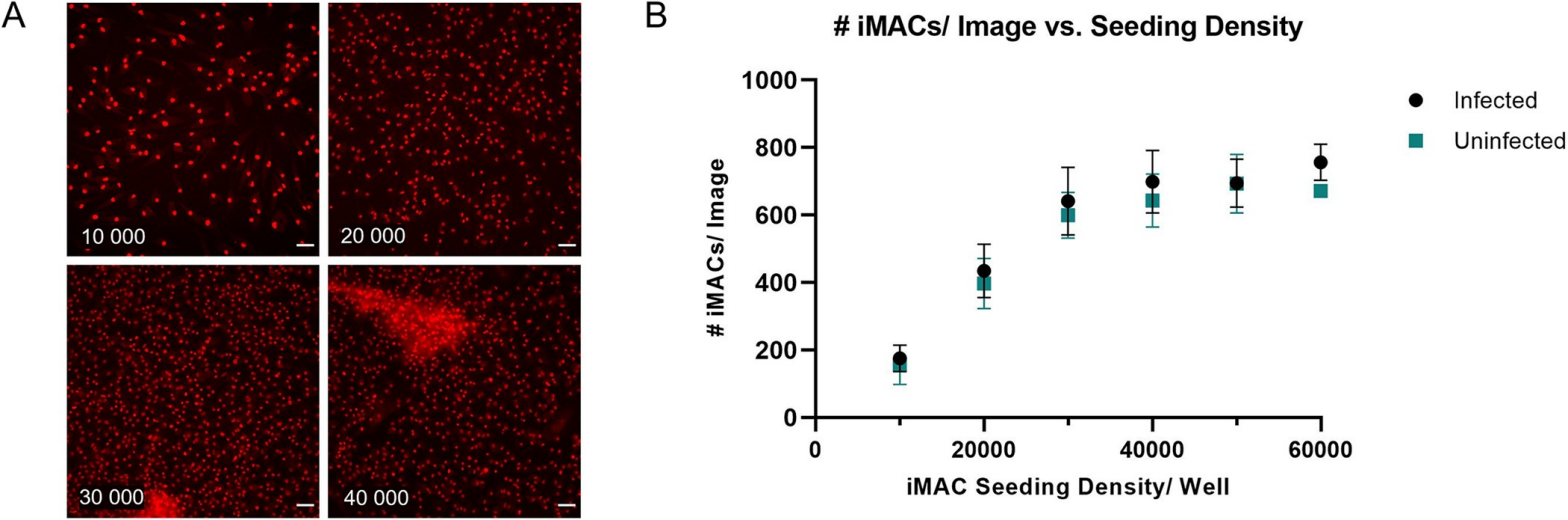
Optimal iMAC seeding density. (A) Representative images of uninfected iMACs seeded at densities of 10,000 to 40,000 cells/well, taken at 20x magnification and stained with DRAQ5. Scale bar = 50 um. (B) Average number of iMACs/ image detected by automated image analysis in function of the initial seeding density for iMACs infected with an MOI of 20 (black) and for uninfected iMACs (blue). N = 4 (independent collection).

In order to obtain infection rates high enough to provide a window for drug screening in 96 well plates, we determined the optimal parameters for an *L*. *donovani* infection model using iMACs as host cells. For this purpose, *L*. *donovani* promastigotes were grown until the late stationary growth phase to obtain a higher proportion of infective metacyclic parasites, which are recognizable by their long and slender appearance (S4A Fig). Late stationary phase was reached after five days of incubation starting with a population of 10^5^ cells/ mL (S4B Fig). For infection, iMACs were seeded at densities of 10,000 to 60,000 iMACs per well and infected with *L*. *donovani*. To test the infection methodology, an MOI of 20 was taken, based on the current in-house infection assay done with PMMs using amastigotes. When the seeding density of iMACs was higher than 20,000 cells per well, there was a significant formation of cell clumps (Fig 2A). This severely interfered with the automated image analysis used for counting the number of iMACs and intracellular parasites. Indeed, with increasing seeding densities, the iMAC count reached a plateau at about 700 iMACs per image when more than 20,000 iMACs were seeded (Fig 2B). The same trend was seen for both infected and uninfected iMACs. Based on these findings, we concluded that the seeding density should not surpass 20,000 iMACs per well, which resulted in an average cell count of 435 ± 80 iMACs per image.

**Fig 3:**
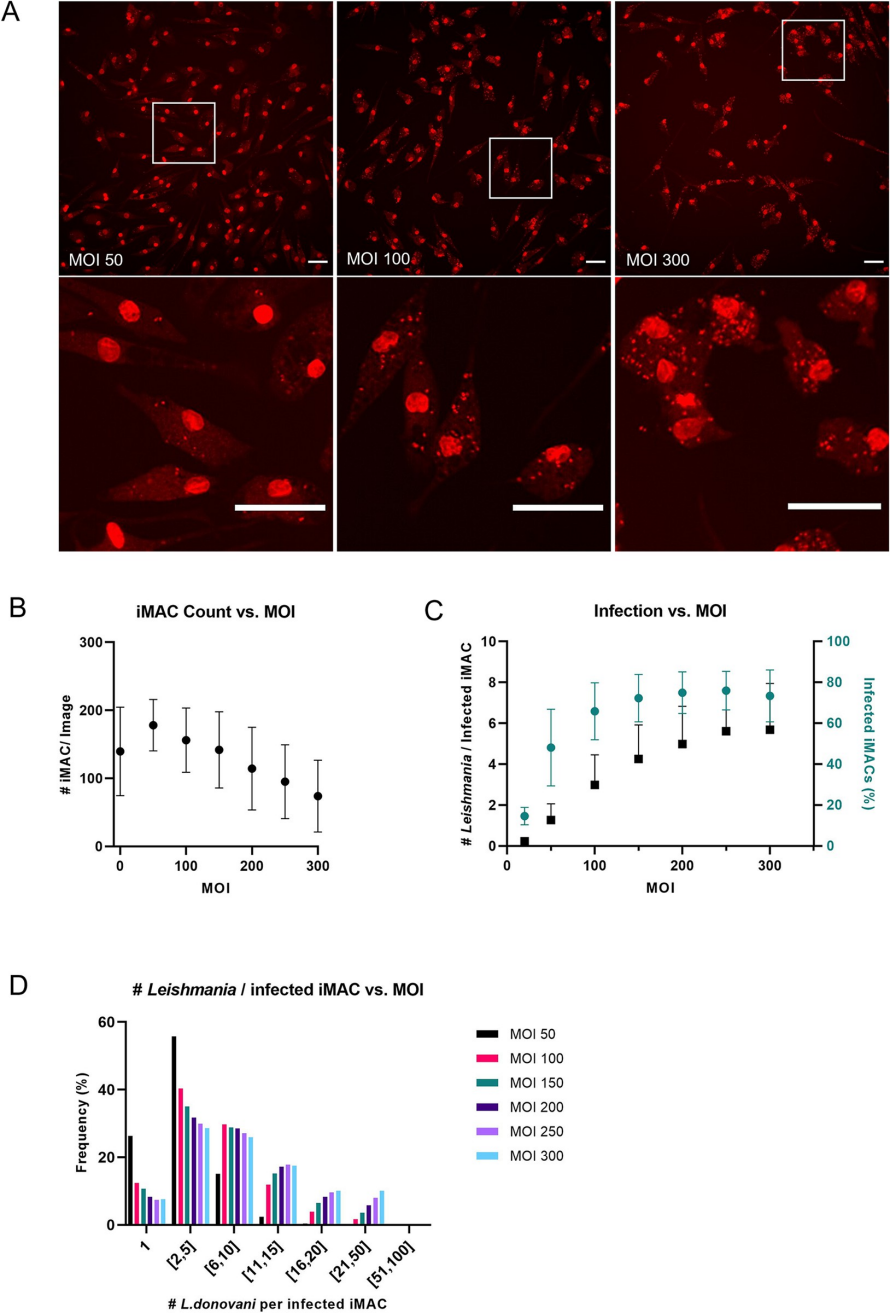
iMAC count and infected iMACs (%) vs. parasite MOI. (A) Representative images of iMACs infected with *L*. *donovani* at a MOI of 50, 100 and 300, taken at 20x magnification and stained with DRAQ5. A higher magnification of the selected region (white box) is presented below. Scale bar = 50 um. (B) Average number of iMACs/ image in function of the MOI. (C) iMAC infection in function of MOI, presented as the number of intracellular parasites per infected iMAC (black) and the percentage of infected iMACs (green). The infection rate was normalized by subtracting the value of the negative control. N = 3 (D) Histogram showing the frequency distribution of number of intracellular *L*. *donovani* per infected iMAC in function of the MOI.

**Fig 4:**
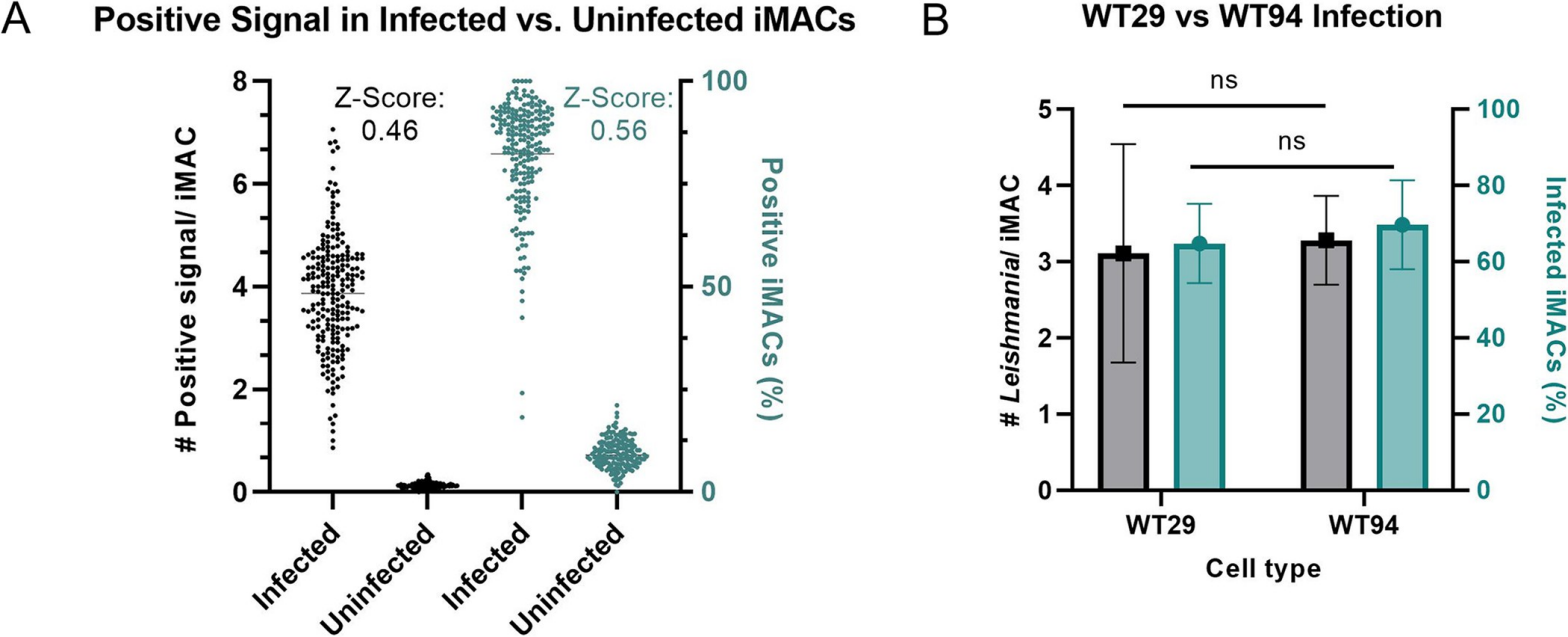
Reproducibility of the model. (A) The number positive dots per iMAC and the percentage of positive iMACs were calculated for both infected and uninfected cells. Each dot represents one image within an assay. From these results corresponding Z-scores were calculated. (B) Infection of WT29 and WT94 iMACs, presented as the number of intracellular parasites per infected iMAC (black) and the percentage of infected iMACs (blue). N = 3 (independent collection). *P<0.05 was considered significant, calculated using unpaired t-tests.

Since an MOI of 20 resulted in low infection rates (14.6 ± 4.3%) (Fig 3C), we tested the infection of iMACs with MOIs between 50 and 300 and assessed the infection rate five days later. We found that as MOI increases, the number of infected iMACs decreased (Fig 3A and 3B). It is currently unclear whether this effect is due to the rupture or simply due to the detachment of infected macrophages. At all MOIs tested, we observed a high infection rate, which plateaued at 75.0 ± 10.2% at an MOI of 200. A similar pattern was found for the number of intracellular parasites, with an average of 5.6 ± 2.0 parasites per infected iMAC, plateauing at an MOI of 250 (Fig 3C). However, a considerable number of cells harboured up to 50 parasites when infected at higher MOIs (Fig 3D). Based on these results, we decided to use an MOI of 100. With this MOI, 65.9 ± 13.9% of iMACs are infected and 82.5% of them have up to 10 *Leishmania* parasites per infected cell. Based on the number of intracellular *Leishmania* parasites per iMAC and the infection rate, we calculated a Z-score of 0.46 and 0.56 respectively for this assay (Fig 4B). To test the robustness of the infection model, iMACs were generated from an additional male iPS line (WT94) and infected at an MOI of 100. No significant difference was found between infection rates of WT29 and WT94 at 64.8 ± 10.4% and 69.7 ± 11.7% respectively (Fig 4A). The average number of *Leishmania* per iMAC was also not significantly different between both cell types. Taken together, these results suggest that iMACs are a suitable model for *L*. *donovani* infection.

### Influence of iMAC age and polarization on infection rate

Once an iMAC-producing culture was established, iMACs could be harvested at each medium change over a period of three to four months. Afterwards the production of the iMACs was markedly reduced and was discontinued. To see if early-time versus late-time iMACs are comparable, the expression of the CD markers and the infection rate were tested at two different time points. The expression of the CD markers (CD14, CD45, CD80, CD86, CD163 and CD206) was comparable between iMACs harvested from young or old cultures, which is consistent with other publications [15] (S5A Fig). To check the iMAC infectibility, iMACs harvested from young (<1.5 months) and old (>2.5 months) EB cultures were infected *with L*. *donovani* at an MOI of 100. Analyses five days after infection could not detect any significant differences between the two conditions (S5B and S5C Fig). The age of the culture therefore appears to have little effect on infection rate.

**Fig 5:**
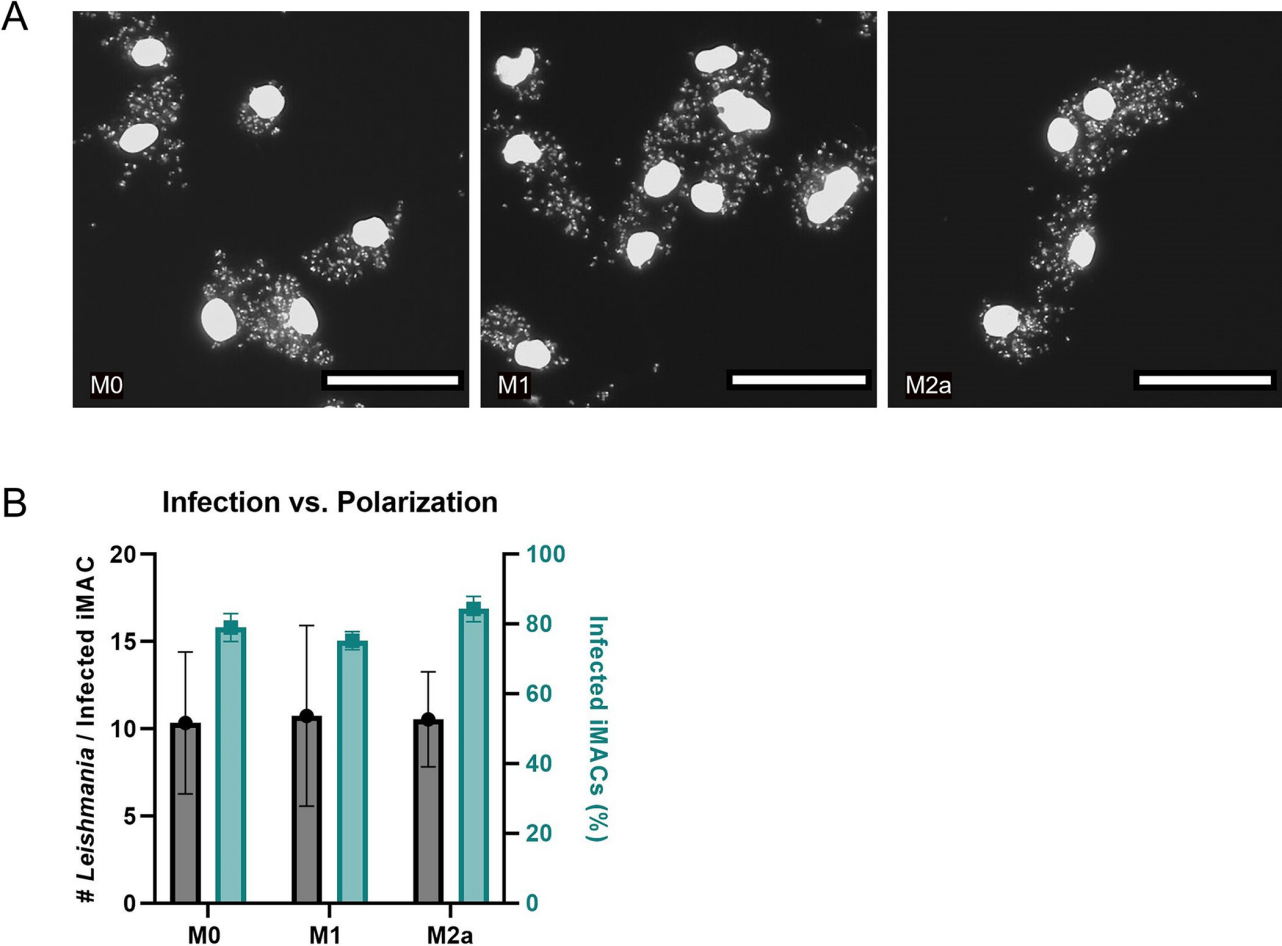
iMAC infectibility vs. Polarization. (A) Representative images of M0, M1 and M2a iMACs, infected with *L*. *donovani* at an MOI of 100, taken at 20x magnification and stained with DAPI. Scale bar = 50 um. (B) iMAC infection in function of the various subtypes, presented as the number of intracellular parasites per infected iMAC (black) or the percentage of infected iMACs (green). The infection rate was normalized by subtracting the value of the negative control. One-way ANOVA analysis showed no significant difference over polarization conditions.

As M1 macrophages are inflammatory while M2 macrophages work anti-inflammatory and help with tissue repair, it is possible that the macrophage subtype influences infectibility of the iMACs with *L*. *donovani*. We polarized M0 macrophages to either the M1 or M2a subtype and infected them with *L*. *donovani* at a MOI of 100. There is no observable difference between the three subtypes five days after infection (Fig 5). Both the number of parasites per infected iMAC and the percentage of infected iMACs were comparable over all conditions (Fig 5).

### Localisation of *Leishmania* in the iMACs

**Fig 6:**
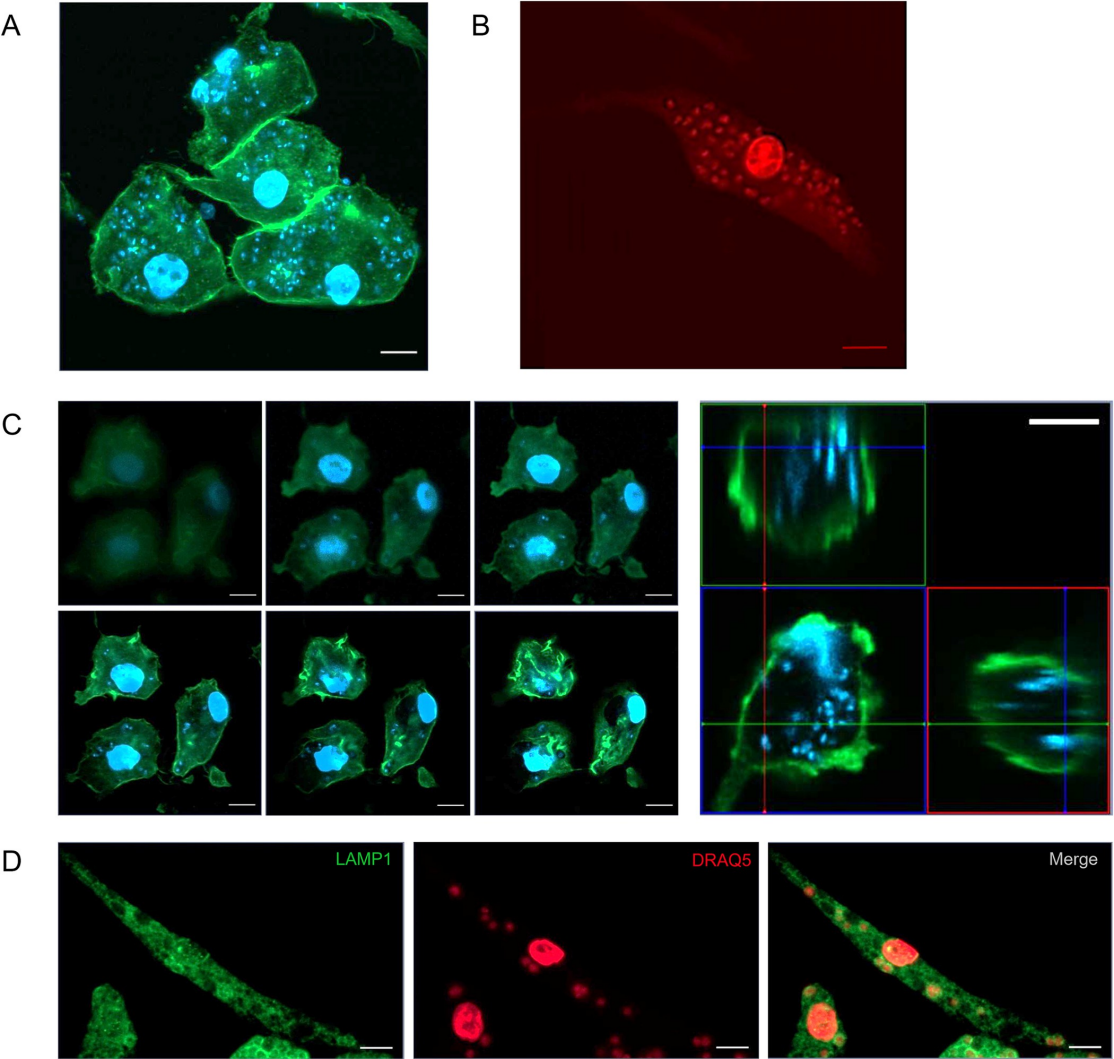
Characterization of iMACs infected with *L*. *donovani*. Infected iMACs were stained with (A) DAPI (blue) and AF488 phalloidin (green) or (B) DRAQ5 (red) (C) Z-stack and intersection of infected iMACs stained with DAPI (blue) and AF488 phalloidin (green) (D) LAMP1 staining of infected iMAC. Images were taken at 64x magnification. Scale bar = 10 um.

Based on these results, we seeded M0 iMACs with a density of 10,000 iMACs per well and infected them with a MOI of 100. Staining was done with a DAPI-phalloidin double stain (Fig 6A) or a DRAQ5 stain (Fig 6B). Both stains allowed a clear visualization of the iMACs and the intracellular *L*. *donovani* parasites. Z-stacking using confocal microscopy clearly showed how the parasites resided within the iMACs and were not e.g. sticking on the outside of the cells (Fig 6C and S1 Video). To determine intracellular localization of the parasites, we stained the infected iMACs with lysosomal-associated membrane protein 1 (LAMP-1) antibodies, a lysosomal marker. Indeed, we observed a localization of *L*. *donovani* inside vacuoles that recruit LAMP-1, pointing towards an intralysosomal localization of the parasites (Fig 6D).

### Testing the iPS-derived system for drug susceptibility

**Fig 7:**
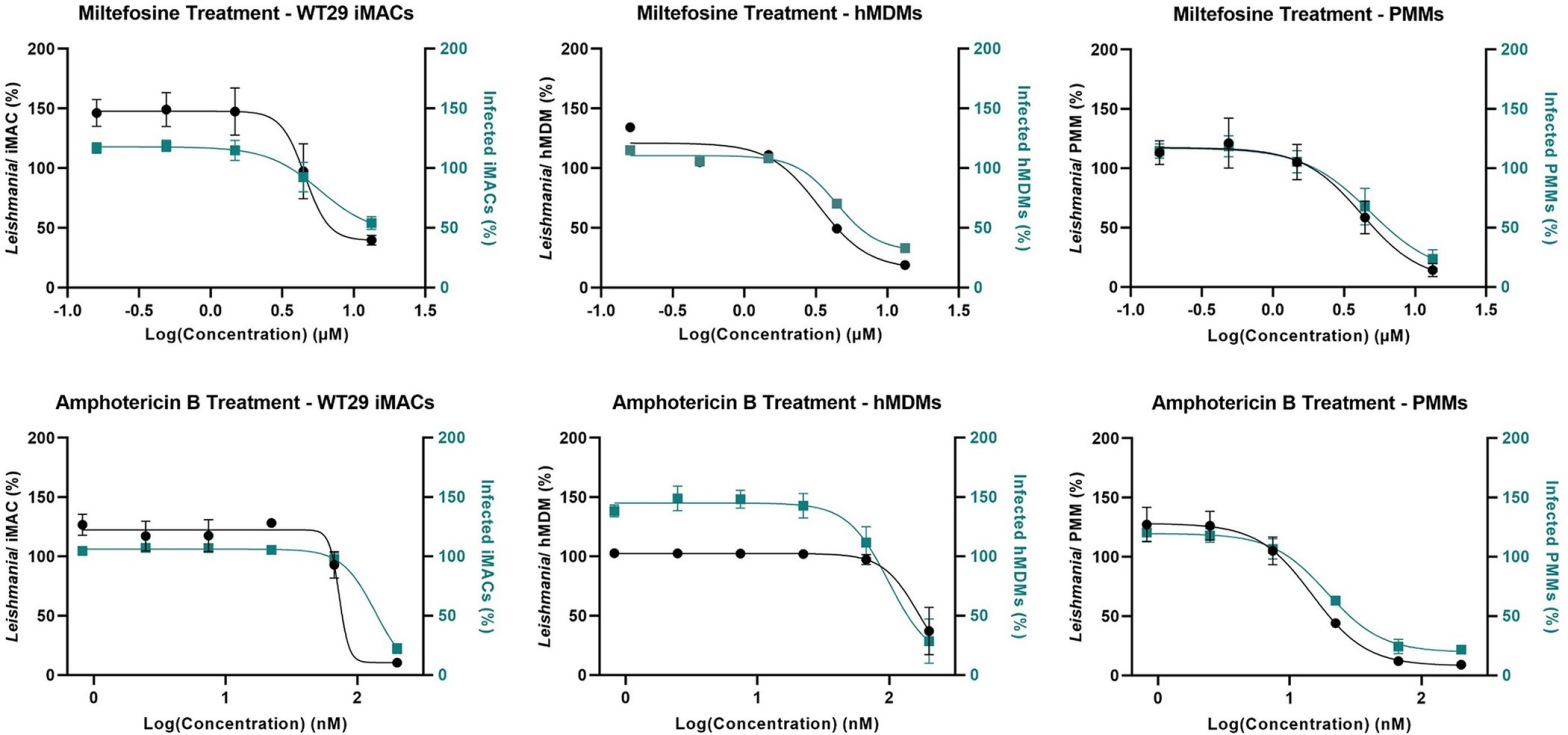
**Drug response for miltefosine and amphotericin B.** Infected WT29 iMACs, PMMs and hMDMs were treated with miltefosine or amphotericin B. The number of intracellular *L*. *donovani* parasites per cell for each concentration is shown on the left y-axis (black). The average number of infected cells is shown on the right y-axis (green). Both parameters are presented as percentages, calculated by correction with an uninfected negative control and normalization with an untreated positive control. N ≥ 3 (independent collection).

Finally, we tested the reference drugs miltefosine and amphotericin B against infected iMACs and calculated the 50% inhibitory concentration (IC_50_) values based on the decrease in infection rate or the decrease of number of intracellular parasites per iMAC. As controls, we also tested these reference drugs against infected PMMs and hMDMs. Cells were treated for four days with miltefosine (0.16 to 40 μM) or amphotericin B (0.82 to 200 nM) 24h after initial infection. As high concentrations of miltefosine are toxic to the macrophages, we left the highest concentration (40 μM) out of the analysis. There was a clear decrease in the number of intracellular parasites over all conditions (Figs 7 and S6). Based on this parameter, the IC_50_ for miltefosine using WT29-derived iMACs as host cells was 3.85 ± 1.90 μM, which is comparable to the IC_50_ values calculated for both the PMM-model (2.80 ± 0.99 μM) and the hMDM model (3.56 ± 0.43 μM) [33] (Table 1). For amphotericin B, we calculated an IC_50_ value of 62.1 ± 39.0 nM using WT29 iMACs as host cells. Amphotericin B showed higher activity in the PMMs (12.8± 4.76 nM) and was slightly less active in the hMDMs (90.93 ± 27.10 nM). Interestingly, the activity of amphotericin B in hMDMs was more comparable to the activity in iMACs than the activity in PMMs. The IC_50_ values calculated based on the percentage of infected cells was higher over all conditions but showed a similar trend. Repeating the same experiment with iMACs generated by iPS line WT94 led to very comparable results (S6 Fig). The IC_50_ values calculated based on the number of intracellular parasites was 3.52 ± 2.28 μM for miltefosine and 58.0 ± 28.2 nM for amphotericin B (Table 1). This high correlation between WT29 and WT94 iMACs and the comparable activity of the reference compounds in iMACs and hMDMs indicate that iPS-derived macrophages could be a valuable tool in the search for new drugs.

**Table 1:** *IC50 values for treatment of WT29 & WT94 iMACs*, *PMMs and hMDMs with miltefosine and amphotericin B*. IC_50_ values were calculated based on the average number of intracellular *L*. *donovani* or the percentage of infected cells. p-values were calculated using one-way ANOVA followed by Tukey’s multiple comparisons test.

	IC_50_ (*L*. *donovani* per cell)	IC_50_ (% infected cells)
	*Miltefosine*	
**WT29**	3.85 ± 1.90 μM	5.83 ± 3.96 μM
**WT94**	3.52 ± 2.28 μM	4.25 ± 2.36 μM
**PMM**	2.80 ± 0.99 μM	5.48 ± 3.47 μM
**hMDM**	3.56 ± 0.43 μM	6.30 ± 3.59 μM
	p = 0.797	p = 0.899
	*Amphotericin B*	
**WT29**	62.1 ± 39.0 nM	105 ± 77.9 nM
**WT94**	58.0 ± 28.2 nM	72.9 ± 40.3 nM
**PMM**	12.8 ± 4.76 nM	19.2 ± 9.35 nM
**hMDM**	90.93 ± 27.10 nM	101.24 ± 41.14 nM
	p = 0.138	p = 0.277

## Discussion

While great advances have been made in automation, high-throughput screening, and high-content readout in anti-leishmanial drug testing in recent decades [17,34,35], the choice of the appropriate host cell for *Leishmania* remained challenging regarding physiology, practicability, price, and ethics. To date, there is no standard infection model used in drug screening for *Leishmania*. Based on our in-house infection assay using PMMs infected with amastigotes, we developed a new infection model using human iMACs as host cells for infection with *L*. *donovani* promastigotes. We used automated image analysis to determine the infectibility of the iMACs and determine the optimal conditions for our model. Here, we show that iMACs are suitable host cells for establishing high infection rates with *L*. *donovani*.

*Leishmania donovani* is an intracellular parasite that lives and replicates in a phagolysosomal compartment [36]. After infection, it induces the formation of parasitophorous vacuoles (PVs), acidic compartments with properties of phagolysosome, in which it develops and replicates. Similar to other models, the intracellular *L*. *donovani* resides in the phagolysosomal compartment of the iMACs as indicated by LAMP-1 staining (Fig 6D) [36,37]. It has been hypothesised that *L*. *donovani* arrests the normal maturation process of the phagosome at a stage before the phagosome reaches its full degradative capacity [38,39]. Because iPS cells are easy to manipulate on the genetic level our model will allow to study the phagosome maturation after *Leishmania* infection in more detail. Interestingly, prepolarization of the iMACs did not influence the infection rate of the cells. In the natural course of infection, alternatively activated (M2) macrophages are more permissible to *Leishmania* infection than the classically activated and inflammatory M1 macrophages [40,41]. In our model, *L*. *donovani* does not have a preference towards M1 or M2 macrophages for establishing infection, however, this aspect deserves more investigation. Possibly, infection with the parasites drives polarization from one state to another.

To test the use of our model for drug screening purposes, we tested the reference drugs miltefosine and amphotericin B against infected macrophages. The activity of these compounds against intracellular *Leishmania* parasites was comparable when using either iMACs, PMMs or hMDMs as host cells. This is also consistent with what has previously been reported using THP-1 cells as host cells for various *Leishmania* spp. [17,33,42,43]. Interestingly, the activity of amphotericin B against infected iMACs and hMDMs was very comparable, while its activity against infected PMMs was slightly higher. We hypothesize that the iMACs are more similar to the hMDMs due to their human origin, which would also make them a more physiological model than mouse macrophages. The sigmoidal curves of the drug responses can vary between replicates (Figs 7 and S6) making it hard to compare them, which is why we focused on the calculated IC_50_ values. It is important to note the differences between IC_50_ values calculated based on the number of intracellular parasites or the number of infected cells, as the latter are consistently higher. Due to the high infection rates, full clearance of the cell is not always reached upon treatment which explains the higher IC_50_ values. Moreover, there is some variation in infection rates between replicates due to culture conditions and we report a large variation in the intracellular number of parasites, often reaching up to 50 parasites per host cell. However, the decrease in number of intracellular parasites can always be measured, resulting in a more stable parameter for calculating the activity of anti-leishmanial compounds.

Although the iMAC model shows promise, the high MOI used is a drawback. Potentially, gradient centrifugation can be implemented to isolate the metacyclic promastigotes or promastigotes can be preconditioned before infection to optimize metacyclogenesis. This could lower the MOI needed to establish high infection rates which could improve this model [44]. Another possibility would be to use amastigotes to establish infection. The use of high-content imaging techniques as described here is also challenging. Our image analysis method is based on the DNA-specific dye DRAQ5 or DAPI, which stains macrophage and parasite nuclei and allows rapid identification and quantification of infected cells. A clear separation based on the size of the parasite’s nucleus, which is several orders of magnitude smaller than the host cell nucleus, allows for efficient quantification of infections. However, inaccuracies in the automated image analysis can either result in an increased false positive or false negative signal. The usage of genetically engineered fluorescent parasites could further improve the model. This would allow for easy identification and quantification of *Leishmania* in infected iMAC. As a genetic modification might impact the virulence of the parasite, usage of parasite-specific antibodies is safer when performing small scale experiments or must be used in validation experiments [45]. However, using fluorescent parasites would enable live cells imaging, which would make is easy to follow the course of infection over time. On top of this, fluorescent parasites would be an important step to perform full genome CRISPR screens to find host proteins required for infection. This has so far been limited due to the technical difficulties involved in genetically manipulating macrophages. While THP-1 cells could be used for this, their malignant origin makes them less reliable as physiological models. Navarro-Guerrero found a way to perform CRISPR screening using lentiviral transfection directly in iMACs [46]. This approach combined with our infection model could potentially identify important regulators of infection.

The next step would be to adapt the system to high-throughput image-based phenotypic screening. The high number of intracellular parasites in our iMAC model opens a great window for performing such screens on a smaller plate format, for example in 384-well or even 1536-well plates. Furthermore, once iPS-technology is established, the differentiation to iMACs is straightforward and results in high yields, reaching up to 10 million cells each week which are easy to isolate. This allows screening of dozens of plates per week, with the limiting factor being the imaging rather than the amount of host cells. While iMACs cannot be frozen for later use, they can be kept at 37°C, 5% CO_2_ for several days after harvesting. With a cost of around 5$/ 1 million iMACs, the price of iMACs is also affordable, making them a feasible alternative to other host cells for high-throughput screening.

As shown, the system allows for intracellular infection and hence future identification of compounds affecting intracellular *Leishmania* replication. Our proof-of-concept experiments with miltefosine and amphotericin B confirmed that targeting intracellular *Leishmania* in iMACs is possible. We believe this host cell model provides an alternative that is more ethical than the usage of PMMs, more practical than hMDMs and more physiological than THP-1 cells. Human iPS-derived macrophages will allow for anti-leishmanial high-throughput screening given their abundant availability and limited genetic variability, and they will facilitate the study of host-parasite interaction at the genetic level.489.

## Supporting information

S1 Fig Automated image analysis. Infected (top) and uninfected cells (bottom) were stained with DRAQ5 and imaged using the CV7000 High Content Imager. CellPathFinder software was used to analyse the images. The original picture (left) is compared with the analysis result (right) showing the identified nuclei (blue), cell bodies (green) and intracellular dots (orange).
(TIF)

S2 Fig CD marker characterisation of iMAC. iMACs were harvested from an 84 day-old EB-culture and stained with various CD markers. Flow cytometry histograms show the expression of the CD markers (blue) compared to an unstained (black) and an isotype (grey) control. The iMAC population was gated based on the FSC-A/ SSC-A plot, followed by gating of the single cells based on the FSC-A/ FSC-H plot. Counts were normalized to the mode.
(TIF)

S3 Fig CD marker characterization of iMACs polarized for five days. iMACs were polarised to the M0 (blue), M1 (IFNγ, purple) or M2a (IL-4, yellow) subtypes for 48h. Flow cytometry histograms show the expression of the cell surface receptors CD14, CD80, CD163 and CD206. Expression is compared to an unstained (black) and an isotype (grey) control. The dotted line marks the intensity peak of the M0 iMACs. The iMAC population was gated based on the FSC-A/ SSC-A plot, followed by gating of the single cells based on the FSC-A/ FSC-H plot and gating of live cells based on the BL2/ FSC-A plot.
(TIF)

S4 Fig Growth characteristics of *Leishmania donovani*. (A) Giemsa staining of late stage stationary promastigotes taken after 5 days in culture (B) Growth curve of *L*. *donovani*, with a starting concentration of 10^5^ parasites/mL, N = 3.
(TIF)

S5 Fig Success of infection vs. EB culture age. (A) iMACs were harvested from old (130 days) and young (30 days) EB-cultures and stained with various CD markers. Flow cytometry histograms shows the expression of the CD markers on iMACs derived from old (blue) and young (yellow) cultures compared to an unstained (black) and an isotype (grey) control. The iMAC population was gated based on the FSC-A/ SSC-A plot, followed by gating of the single cells based on the FSC-A/ FSC-H plot. Counts were normalized to the mode. (B) iMACs harvested from young (< 1.5 months) or old (>2.5 months) EB-cultures were infected with *L*. *donovani*, stained with DAPI. Scale bar = 50 μm. (C) iMAC infection for iMACs derived from young vs. old EB-cultures, presented as either number of *Leishmania* per iMAC (grey) or percentage of infected iMACs (blue). *P<0.05 was considered significant, calculated using unpaired t-tests.
(TIF)

S6 Fig Drug response for miltefosine and amphotericin B in WT94 iMACs. Infected WT94 iMACs were treated with miltefosine or amphotericin B. The number of intracellular *L*. *donovani* parasites per cell for each concentration is shown on the left y-axis (black). The average number of infected cells is shown on the right y-axis (green). Both parameters are presented as percentages, calculated by correction with an uninfected negative control and an untreated positive control. N ≥ 3 (independent collection).
(TIF)

S1 Video Intracellular localization of *L*. *donovani* inside the iMACs. Infected iMACs were stained with DAPI (blue) and AF488 phalloidin (green). A Z-stack of the cells was obtained and presented as a video. Images were taken at 64x magnification. Scale bar = 10 um.
(GIF)
