# Supplementary figures and images for: Induced pluripotent stem cell-derived human macrophages as an infection model for Leishmania donovani
Source: PLoS Negl Trop Dis. 2024 Jan 2;18(1):e0011559. doi: 10.1371/journal.pntd.0011559 (PMC10786377; doi:10.1371/journal.pntd.0011559)

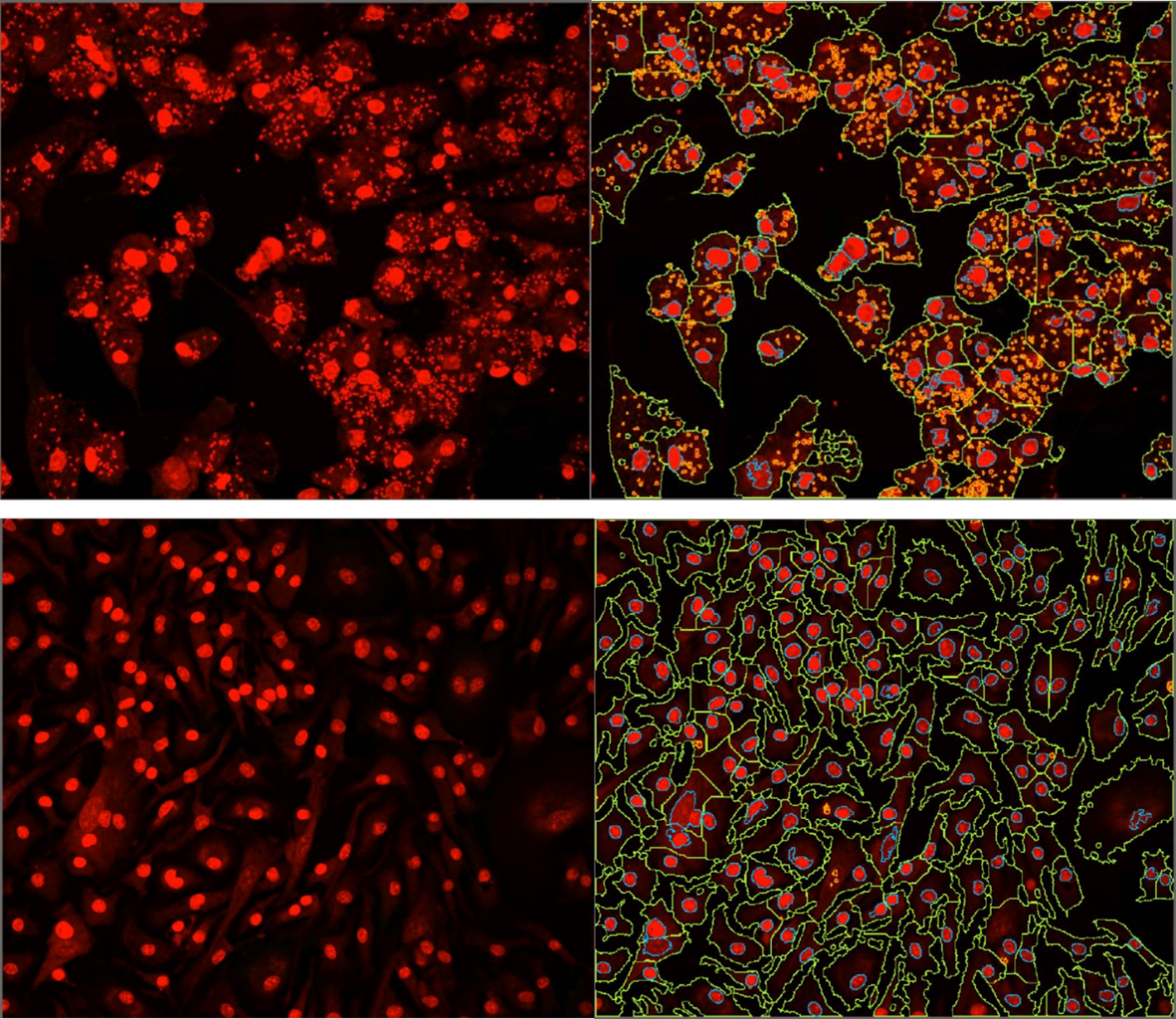

Supplement: S1 Fig — Infected (top) and uninfected cells (bottom) were stained with DRAQ5 and imaged using the CV7000 High Content Imager. CellPathFinder software was used to analyse the images. The original picture (left) is compared with the analysis result (right) showing the identified nuclei (blue), cell bodies (green) and intracellular dots (orange). (TIF) [file pntd.0011559.s001.tif]

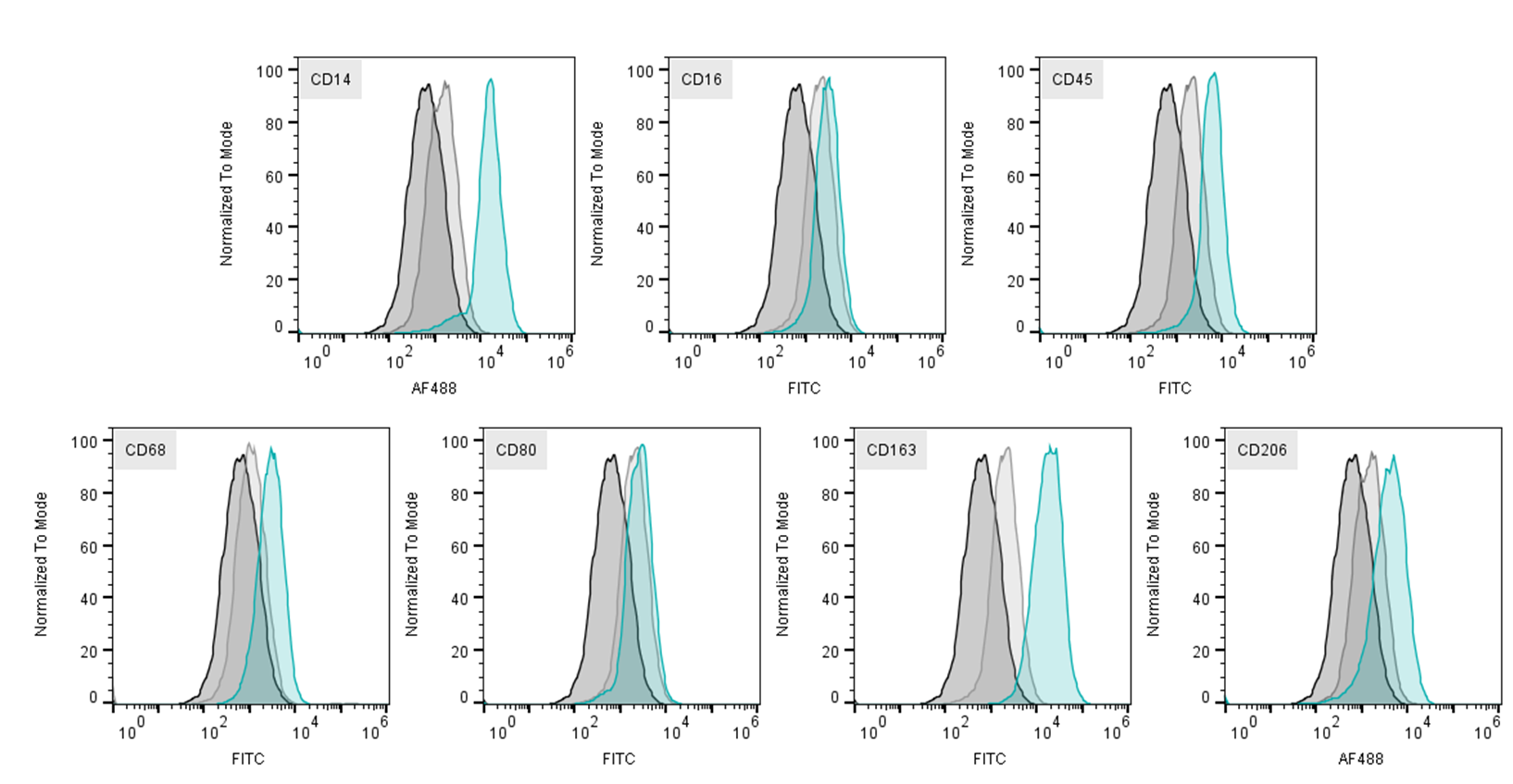

Supplement: S2 Fig — iMACs were harvested from an 84 day-old EB-culture and stained with various CD markers. Flow cytometry histograms show the expression of the CD markers (blue) compared to an unstained (black) and an isotype (grey) control. The iMAC population was gated based on the FSC-A/ SSC-A plot, followed by gating of the single cells based on the FSC-A/ FSC-H plot. Counts were normalized to the mode. (TIF) [file pntd.0011559.s002.tif]

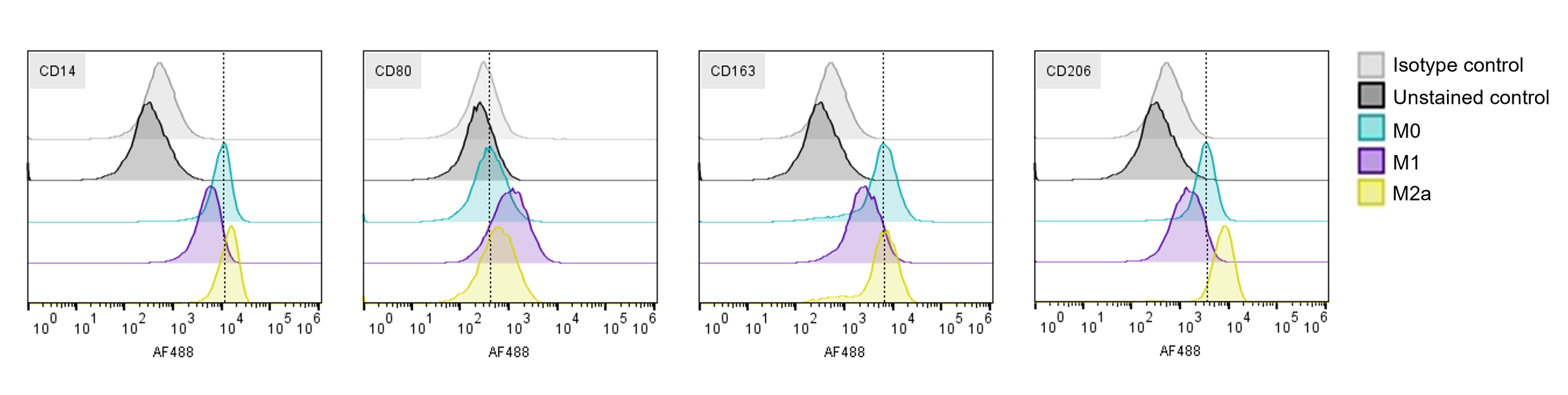

Supplement: S3 Fig — iMACs were polarised to the M0 (blue), M1 (IFNγ, purple) or M2a (IL-4, yellow) subtypes for 48h. Flow cytometry histograms show the expression of the cell surface receptors CD14, CD80, CD163 and CD206. Expression is compared to an unstained (black) and an isotype (grey) control. The dotted line marks the intensity peak of the M0 iMACs. The iMAC population was gated based on the FSC-A/ SSC-A plot, followed by gating of the single cells based on the FSC-A/ FSC-H plot and gating of live cells based on the BL2/ FSC-A plot. (TIF) [file pntd.0011559.s003.tif]

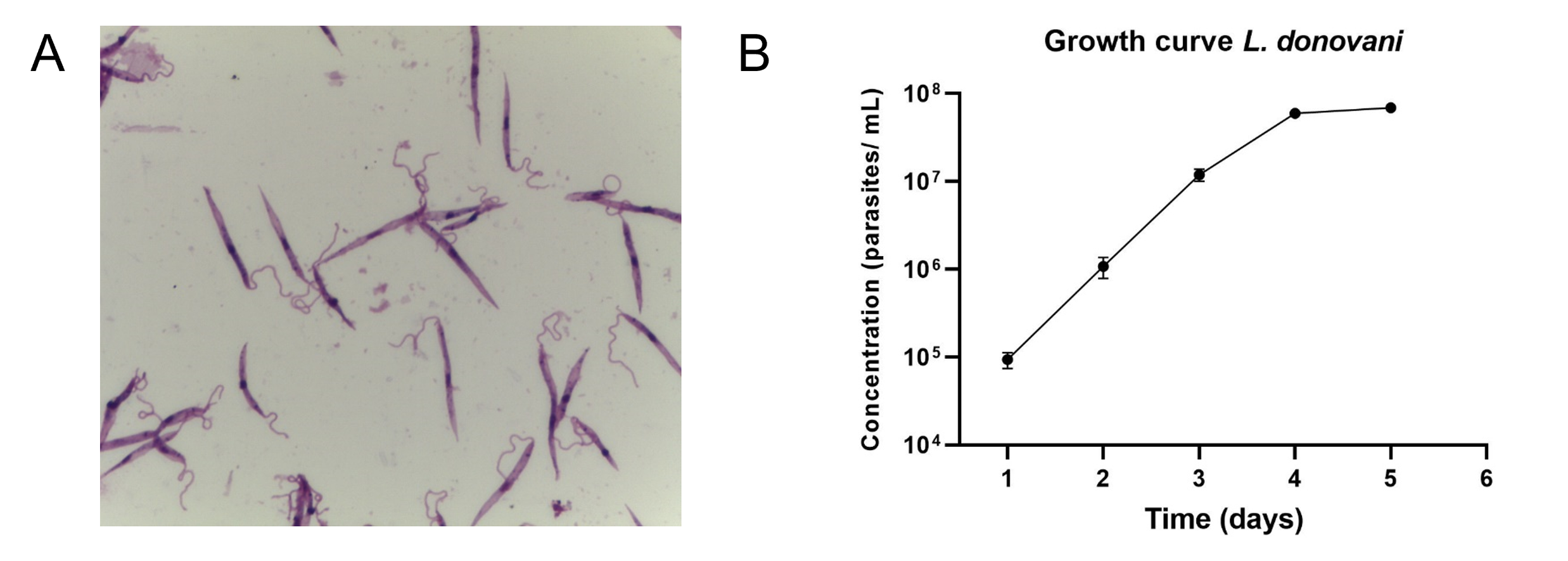

Supplement: S4 Fig — (A) Giemsa staining of late stage stationary promastigotes taken after 5 days in culture (B) Growth curve of L. donovani, with a starting concentration of 105 parasites/mL, N = 3. (TIF) [file pntd.0011559.s004.tif]

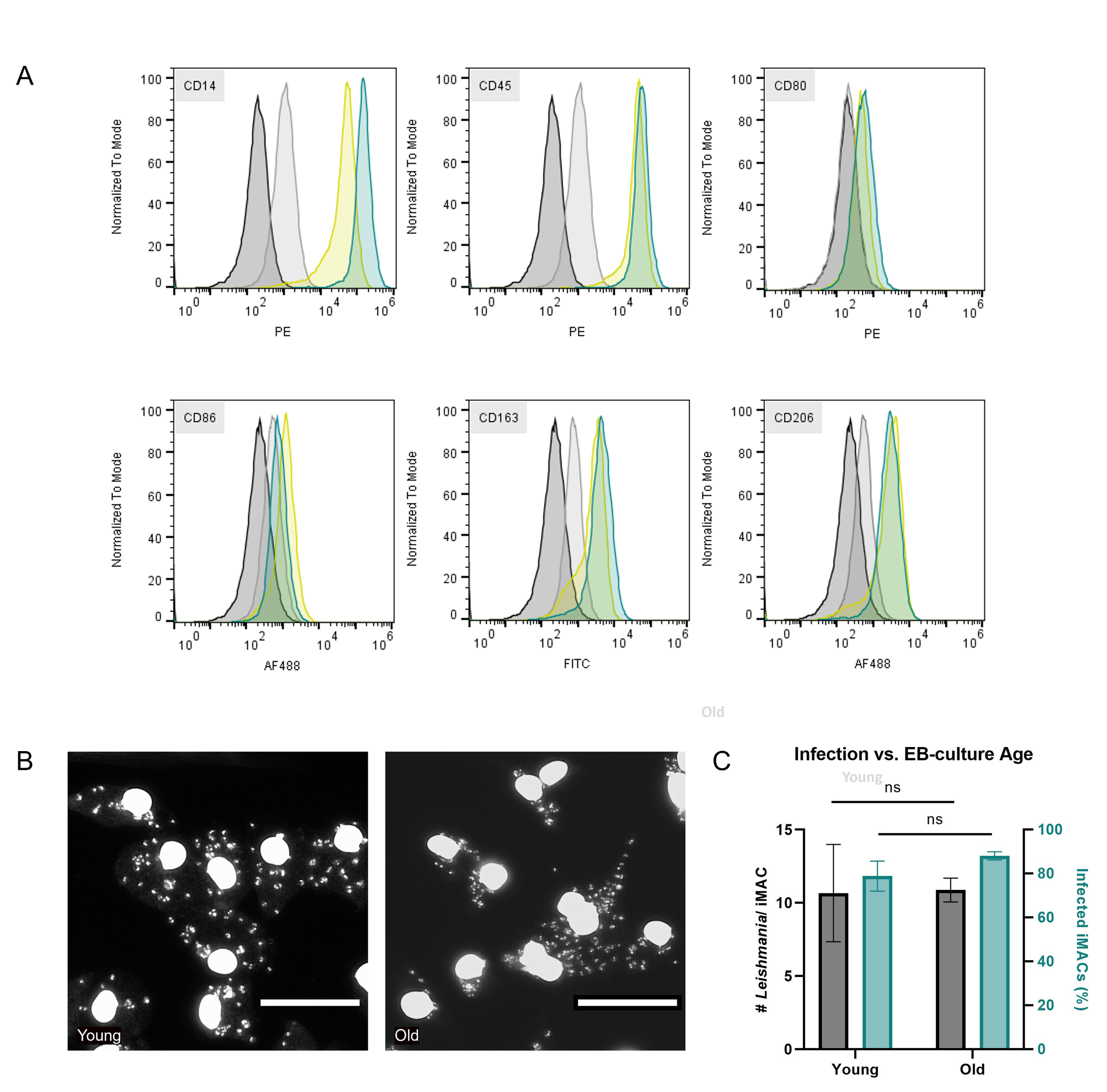

Supplement: S5 Fig — (A) iMACs were harvested from old (130 days) and young (30 days) EB-cultures and stained with various CD markers. Flow cytometry histograms shows the expression of the CD markers on iMACs derived from old (blue) and young (yellow) cultures compared to an unstained (black) and an isotype (grey) control. The iMAC population was gated based on the FSC-A/ SSC-A plot, followed by gating of the single cells based on the FSC-A/ FSC-H plot. Counts were normalized to the mode. (B) iMACs harvested from young (< 1.5 months) or old (>2.5 months) EB-cultures were infected with L. donovani, stained with DAPI. Scale bar = 50 μm. (C) iMAC infection for iMACs derived from young vs. old EB-cultures, presented as either number of Leishmania per iMAC (grey) or percentage of infected iMACs (blue). *P<0.05 was considered significant, calculated using unpaired t-tests. (TIF) [file pntd.0011559.s005.tif]

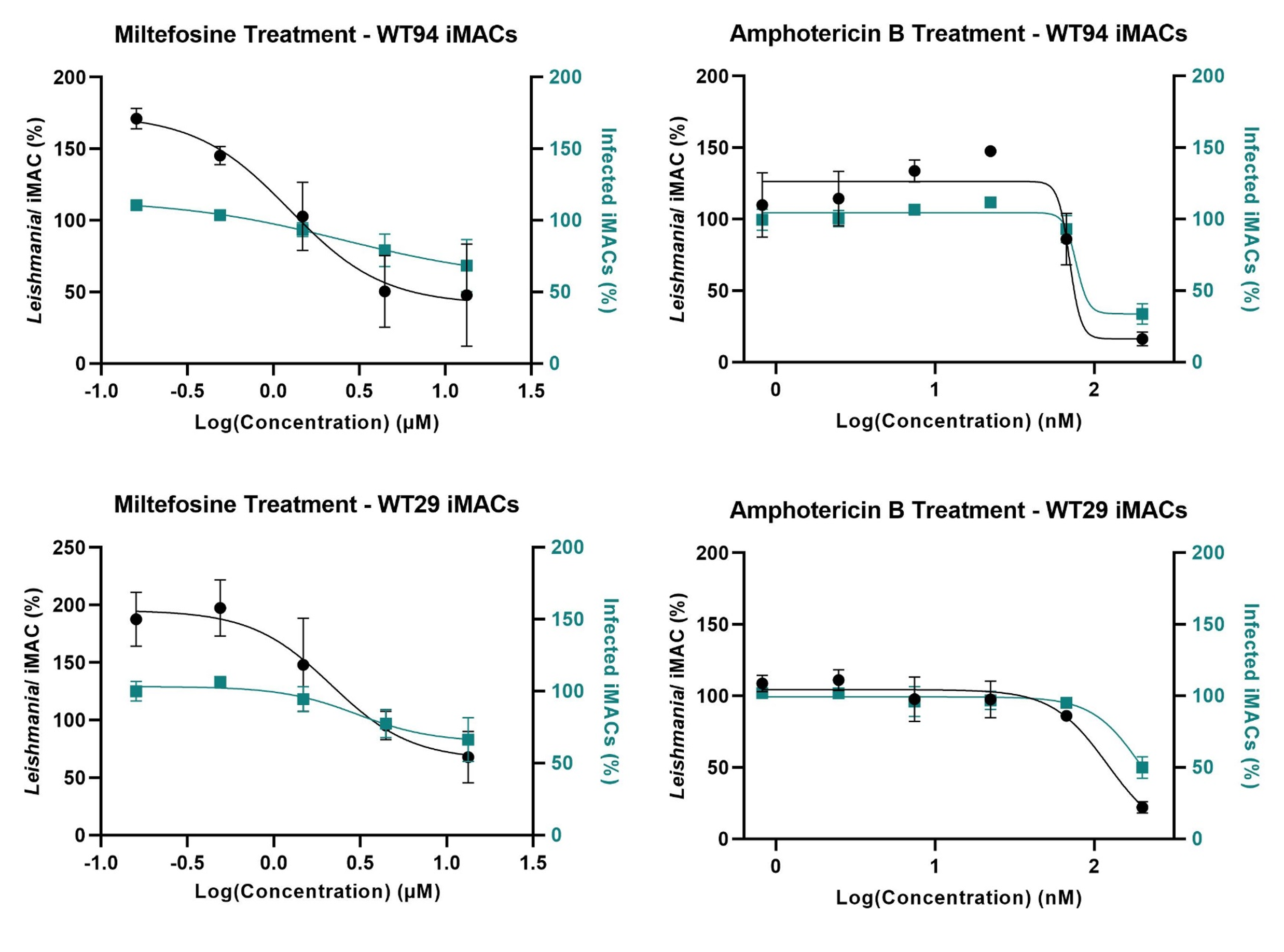

Supplement: S6 Fig — Infected WT94 iMACs were treated with miltefosine or amphotericin B. The number of intracellular L. donovani parasites per cell for each concentration is shown on the left y-axis (black). The average number of infected cells is shown on the right y-axis (green). Both parameters are presented as percentages, calculated by correction with an uninfected negative control and an untreated positive control. N ≥ 3 (independent collection). (TIF) [file pntd.0011559.s006.tif]

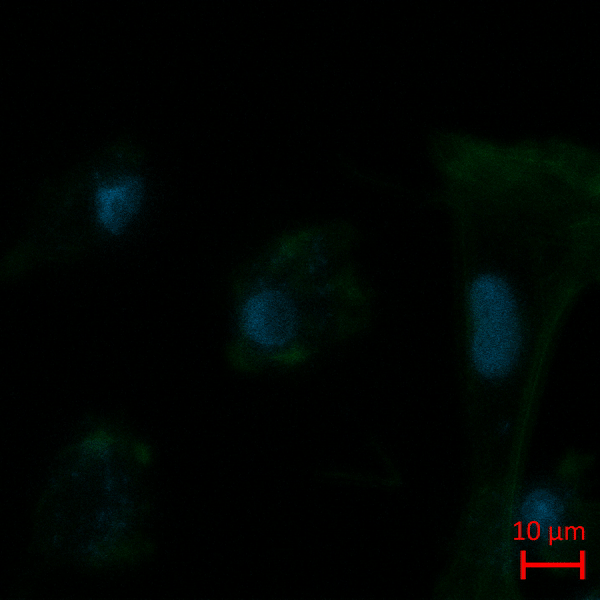

Supplement: S1 Video — Infected iMACs were stained with DAPI (blue) and AF488 phalloidin (green). A Z-stack of the cells was obtained and presented as a video. Images were taken at 64x magnification. Scale bar = 10 um. (GIF) [file pntd.0011559.s007.gif]
